# A multifaceted comparison between the fruit-abscission and fruit-retention cultivars in ornamental crabapple

**DOI:** 10.3389/fpls.2022.1013263

**Published:** 2022-09-21

**Authors:** Xue Wang, Yi Wang, Shufang Yan, Xuan Sun, Hongyan Liu, Beibei Cheng, Xingxing Xu, Zunzheng Wei, Guojun Zhang

**Affiliations:** ^1^College of Horticultural Science and Technology, Hebei Key Laboratory of Horticultural Germplasm Excavation and Innovative Utilization, Hebei Normal University of Science and Technology, Qinhuangdao, China; ^2^Institute of Grassland Flowers and Ecology, Beijing Academy of Agriculture and Forestry Sciences, Beijing, China; ^3^College of Horticulture, China Agricultural University, Beijing, China; ^4^Hebei Academy of Forestry and Grassland Sciences, Hebei Forest City Construction Technology Innovation Center, Shijiazhuang, China

**Keywords:** ornamental crabapple, fruit abscission, abscission zone, cell wall hydrolase, transcriptomics

## Abstract

The ornamental crabapple is a multipurpose landscaping tree that bears brilliant fruit throughout the winter. However, whether or not its fruit persists after maturation is specifically correlated to cultivar characteristics. In this work, we screened two different types that display fruit-retention (“Donald Wyman,” “Red Jewel,” and “Sugar Tyme”) and fruit-abscission (“Radiant” and “Flame”) in Northern China across the whole winter using multi-year successional records. Fruit-abscission was determined predominantly by the abscission zone established at the base of the pedicel, regardless of fruit size and pedicel length, according to the results of the comparative research. The primary physiological rationale was the accumulation of hydrolases activity (pectinesterase, cellulase, polygalacturonase, and β-glucosidase). Comparative transcriptomics further identified a number of upregulated DEGs involved in the synthesis pathways of canonical phytohormones, such as ethylene, jasmonic acid, abscisic acid, and cytokinin, as well as 12 transcription factors linked in downstream signaling in fruit-abscission cultivars. Finally, a model incorporating multi-layered modulation was proposed for the fruit abscission of ornamental crabapple. This study will serve as the foundation for the development of fruit-viewing crabapples that have an extended ornamental lifetime.

## Introduction

The ornamental crabapple (*Malus* crabapple) is a superb attractive tree owing to its vividly colored flowers, foliage, and fruits. Currently, the majority of researches are devoted to these features (Goncharovska et al., 2022). Even so, the ornamental crabapple’s ability to sustain fruit after blooming is always highlighted as the demand for gardening increases exponentially around the world. The time of fruit abscission, which is a significant determinant of aesthetic value, varies substantially across the present cultivars. While some cultivars of ornamental crabapple lose their fruit in September, other may keep theirs until the next flowering season. Several species, including apple (Heo and Chung, 2019), litchi (Zhao and Li, 2020), pear (Li et al., 2019), and olive (Parra et al., 2020), have had their molecular processes of fruit abscission investigated. However, the internal mechanism of fruit abscission of ornamental crabapple has not been examined. Hence, it is crucial to explore the effect of fruit abscission on ornamental crabapple.

The now-recognized abscission process normally involves four stages (Estornell et al., 2013; Tucker and Kim, 2015; Patharkar and Walker, 2018). The process starts with the differentiation of abruption layers. Several layers of stratified cells are formed before to abscission. Abscission responses to the phytohormone signals constitute the second step (such as decrease in auxin and increase in ethylene). The third stage involves modifying the cell wall and separating the cells. Various enzymes are produced at this step, resulting in the degradation of pectin and polysaccharides in the cell wall. Cell adhesion is eliminated by the degrading of pectin and cellulose by cell wall hydrolase (Zamil and Geitmann, 2017). In grape, cellulase (CX) and polygalacturonase (PG) synthesis is enhanced in the abscission zone (AZ), which facilitates fruit abscission (Wu et al., 2021). Analysis of gene expression in the AZ of litchi small fruit demonstrated that the expression of *LcPG1* is positively related to fruit abscission (Peng et al., 2013). The fourth stage is characterized by fruit abscission differentiation and the formation of a protective layer. The fact that some of the laminate cells are degraded by cell hydrolase and lose adhesion, others form protective layers.

It has been shown that fruit abscission is a highly coordinated biochemical process (Sun et al., 2009). Recent studies have demonstrated that senescence and the fruit abscission process are influenced by a range of elements, hormone being one of the most prominent (Agusti et al., 2012; Gulfishan et al., 2019). According to prior research, ethylene (ET), abscisic acid (ABA), and cytokinin (CTK) may stimulate fruit abscission, but auxin (IAA) and gibberellin (GA) can inhibit fruit abscission (Taylor and Whitelaw, 2001). *MdACS5A* and *MdACO1* in the mature fruit AZ may be associated with the physiological fruit abscission of “Golden delicious” and “Fuji” prior to harvest (Li et al., 2010). Apple fruit abscission and enhanced Acrylonitrile-butadiene-acrylate (ABA) biosynthesis are related with the stimulation of 9-cis-epoxide carotenoid dioxygenase gene that is sensitive to ABA (Giulia et al., 2013). Grape IAA and GA regulate auxin homoeostasis by inhibiting polar auxin transport in order to control abscission. Reduced transcription of four *VvPIN* genes inhibits polar IAA transport, leading to fruit abscission (Kuhn et al., 2016). In apple, the inactive gene of cytokinin dehydrogenase (CKX) was upregulated during fruit shedding induction, indicating that CTK was reduced during this process (Botton et al., 2011). In conclusion, fruit abscission is mediated by the coordinated action of several hormones.

Here, we examined fruit-abscission and fruit-retention cultivars of ornamental crabapple, which, respectively, shed their fruits in the fall and the following spring. Wood slicing technology, RNA-seq, and quantitative real-time PCR (qRT-PCR) were used to compare the two and explore the internal processes determining fruit abscission in ornamental crabapple. This study offered a framework for future research on the morphology, anatomy, and molecular biology of organ abscission in ornamental crabapple and provided the foundation for uncovering the molecular mechanism of organ abscission in ornamental crabapple and developing cultivars with a prolonged ornamental lifecycle.

## Materials and methods

### Plant materials

In 2011, ornamental crabapple cultivars including “Donald Wyman,” “Red Jewel,” “Sugar Tyme,” “Radiant,” and “Flame” were imported from the United States. The grafted seedlings were transplanted to the resource garden at Hebei Normal University of Science & Technology (39°N, 119°E). Each cultivar was planted with five replications, as well as the block layout was completely random. After they were flowered in 2013, the fruit growth was monitored for 5 years, including the fruit ornamental period, the abscission period, and the abscission position on the dwarf shoot.

Depending on whether the fruit falls off in autumn, these ornamental crabapples may be divided into two groups (Supplementary Figure 1A). “Donald Wyman,” “Red Jewel,” and “Sugar Tyme” are described as fruit-retention cultivars. They retain fruits well throughout the winter, even until March or April of the following year. “Radiant” and “Flame” are fruit-abscission cultivars. Their fruit gradually falls in late September. The AZ between the fruit pedicel and dwarf shoot is more easily to form in fruit-abscission cultivars than fruit-retention cultivars (Supplementary Figure 1B).

### Fruits related traits descriptions in ornamental crabapple

According to our previous investigations (Supplementary Table S1), the end of September is a critical time for determining whether ornamental crabapple fruit will fall off. On September 25, 2019, 20 healthy fruits per cultivar were chosen at random from perennial shoots. Trait parameters defined using Guideline for the Conduct of Tests for Distinctness, Uniformity, and Stability-Ornament Apple (UPOV:TG/192/1). These characteristics comprised the size of fruit and pedicel. The size of fruit includes its length, width, and weight, whereas the size of fruit pedicel includes its length and thickness. The length and width of the fruit, as well as the length and thickness of the fruit pedicel for each individual fruit, were measured using vernier caliper. In addition, a 1–2 cm piece of dwarf shoot and fruit pedicel was cut with a knife and then divided into three parts for extensive evaluation. One part was fixed in a mixture of formaldehyde, acetic acid, and ethanol (FAA) for tissue sectioning, while the other two parts were frozen in liquid nitrogen and stored at −80°C for enzymatic activity assays and RNA extraction.

### Anatomical observation of fruit pedicel AZ

Those samples fixed in FAA for 24 h initially were sectioned longitudinally and transversely to a thickness of approximately 8 μm using a microtome. Then, the slices were positioned on the slides. These slides were finally treated with the following reagents in sequence: 1.0% safranin solution, 50% ethanol, 70% ethanol, 85% ethanol, 0.1% solid green solution, 100% ethanol, and resin sealing. These were finally inspected with an Olympus DP 72 light microscope.

### Enzyme extraction and activity assay

Pectinesterase (PE), CX, PG, and β-glucosidase (BG) were assayed in accordance with the manufacturer’s instructions (Suzhou Keming Biotechnology). Initially, 0.05 g of frozen shoot and fruit pedicels were pulverised with 200 ml of a pretreatment solution in an ice-bath mortar. The supernatant containing the enzyme was then retrieved by centrifuging at 16,000 *g* for 10 min at 4°C. The manufacturer’s kit outlines the methods available for the specific examination of each enzyme. The enzyme activity of the PE was examined using titration, while the activities of the other three enzymes were examined using an Enzyme-Linked Sorbent Assay (ELSA), with absorbance values recorded at 620, 540, and 400 nm, respectively. There were three replicates for each sample.

### cDNA library preparation and RNA sequencing

RNA extraction was performed using the RNAprep pure Plant Kit (Beijing Tian gen Co., Ltd.). RNA purity and integrity were examined using an Agilent 2100 Bioanalyzer. The cDNA was synthesized with a Thermo Frist Strand cDNA Synthesis Kit (Thermo Fisher Scientific). Using Illumina Truseq RNA Sample Prep Kits (Illumina, Santiago), five cDNA libraries each with roughly 130–150 bp insertion fragments were generated, and subjected to paired-end sequencing on an Illumina HiSeq platform owned by Beijing Biomarker Technologies Co., Ltd. All raw data were deposited to the Genome Sequence Archive (GSA) with the accession number CRA004670.

### Sequence assembly, unigenes functional annotation and transcription factor identification

After quality control, we acquired the clean reads for each cultivar and assembled them into non-redundant unigenes using the Trinity platform embedded on the website (http://www.biocloud.net/, Beijing Biomarker Technologies Co., Ltd). These unigenes served as reference repository to examine the variations in gene expression among five cultivars. In addition, these assembled non-redundant unigenes were functionally blasted against a number of databases, including Non-Redundant Protein Sequence (NR), Gene Ontology (GO), Clusters of Orthologous Groups for Eukaryotic Complete Genomes (KOG), Kyoto Encyclopedia of Genes and Genomes (KEGG), Clusters of Orthologous Groups (COG), and Swiss-Prot, among others. Furthermore, homology search for non-redundant unigenes against plant transcription factor databases (Plant TFDB) identified the transcription factor using iTAK software with a significant cut-off of E-value (10E−5; Zheng et al., 2016).

### Analysis of gene differential expression

Consensus unigenes of five cultivars were used for the analysis of differentially expressed genes. The EBSeq software (Leng et al., 2013) was used to identify the set of differentially expressed genes (DEGs) among five ornamental crabapples cultivars. The DEGs were screened using the Benjamini-Hochberg-corrected. The false discovery rate (FDR) was as a key indicator. Those consensus unigenes with more than two-fold differential expression and FDR less than 0.01 were considered candidate DEGs.

To calculate consensus unigene expression levels, the number of uniquely aligned fragments was normalized using the FPKM (Fragments Per Kilobase of exon model per Million mapped fragments) value (Mortazavi et al., 2008).

### Quantitative PCR analysis

Total RNA extraction and cDNA synthesis for each sample were conducted as in previous RNA library preparation. Candidate genes in the qRT-PCR examination included *McPME12* (Pectinesterase 12), *McBGLU12* (β-glucosidase 12), *McCEL1* (Cellulase 1), *McPG* (Polygalacturonase), *McbHLH75* (Basic helix–loop–helix domain 75), and *McWRKY75* (WRKY transcription factor 75). The gene-specific PCR primers designed by Primer Premier 5 were presented in Supplementary Table S5. The *McActin* gene was used as an internal reference gene. The relative expression levels of genes were quantified using ABI StepOne Plus equipment. Each sample comprises three biological replicates. Relative expression levels for each gene were analyzed using the 2^ ^(−∆∆Ct)^ method (Livak and Schmittgen, 2001).

### Statistical analysis

All data were analyzed using the IBM SPSS Statistics 20.0 software (SPSS Inc., United States). Significance and multiple comparisons in each cultivar were assessed using ANOVA and Tukey’s multiple range test with a significance threshold of 0.05.

## Results

### Effects of fruit-related traits of ornamental crabapple on fruit abscission

Our prior sequential field observations spanning 5 years suggested that the five cultivars’s fruit-shedding periods varied substantially. The fruit abscission phase of “Donald Wyman,” “Red Jewel,” and “Sugar Tyme” occurred normally in mid-March of the following year. “Radiant” dropped all of its fruit in early October, while “Flame” shed sooner, virtually all of its fruit by the end of September. Since fruit and fruit pedicel characteristics are major determinants of fruit drop, we evaluated the fluctuations in these two related parameters on September 25 for each cultivar. The changes in the two assessed fruit-related variables were showed in Table 1.

**Table 1:** Fruit-related traits description in fruit-retention and -abscission cultivars of ornamental crabapple.

Cultivars		Fruit size		Fruit pedicel		
		Length (mm)	Width (mm)	Weight (g)	Length (mm)	Thickness (mm)
Fruit-retention cultivars	Donald Wyman	11.09 ± 0.90 bc	11.42 ± 0.95b	1.02 ± 0.18b	36.10 ± 5.64ab	0.63 ± 0.12ab
	Red Jewel	10.33 ± 0.70c	9.99 ± 0.56b	0.82 ± 0.14b	31.69 ± 3.31bc	0.43 ± 0.08 cd
	Sugar Tyme	12.37 ± 1.18b	10.62 ± 0.83b	1.06 ± 0.27b	27.91 ± 3.40c	0.34 ± 0.05d
Fruit-abscission cultivars	Radiant	11.43 ± 0.60bc	11.47 ± 1.22b	1.05 ± 0.33b	39.11 ± 5.75a	0.53 ± 0.08bc
	Flame	18.73 ± 1.68a	16.83 ± 2.09a	3.42 ± 0.95a	20.76 ± 3.14d	0.66 ± 0.13a

Fruit-retention cultivars, including “Donald Wyman,” “Red Jewel,” and “Sugar Tyme,” featured fruit length, width, and weight, respectively, of 10.33–12.37 and 9.99–11.42 mm, and 0.82–1.06 g, while fruit-abscission cultivars, “Radiant” and “Flame,” with 11.43–18.73 and 11.47–16.83 mm, and 1.05–3.42 g, respectively (Table 1). “Flame” as a cultivar with early fruit abscission possibly connected with its largest fruit (18.73 mm in length and 16.83 mm in width, respectively) and greatest weight (3.42 g). Even though “Radiant” also lost it fruit earlier in October, its average fruit weight of 1.05 g is comparable to that of fruit-retention cultivars, such as “Doanld Wyman” (1.02 g), “Red Jewel” (0.82 g), and “Sugar Tyme” (1.06 g). Those suggested that fruit size and weight may not be the primary factors of early fruit abscission in “Radiant.” The length and thickness of fruit pedicels in the fruit-retention and -abscission cultivars, were 27.91–36.10 and 0.34–0.63 mm, as well as 20.76–39.11 and 0.53–0.66 mm, respectively. The fruit pedicels of the cultivar “Flame” were the shortest (20.76 mm) but the thickest (0.66 mm), while those in the “Radiant” were the longest (39.11 mm) but relatively thicker (0.53 mm). It is rather unexpected that “Donald Wyman” has both the longest (36.10 mm) and thickest (0.63 mm) fruit pedicels. This suggests that fruit abscission is not directly associated with any of these fruit pedicel characteristics. Fruit size and fruit pedicel variation did not change significantly between fruit-retention and -abscission cultivars, according to our results. This is also indicative of the fact that the complex attribute of fruit abscission is substantially determined by the underlying processes specific to the cultivars.

### Microstructure observation on fruit AZ in ornamental crabapple

**Figure 1:**
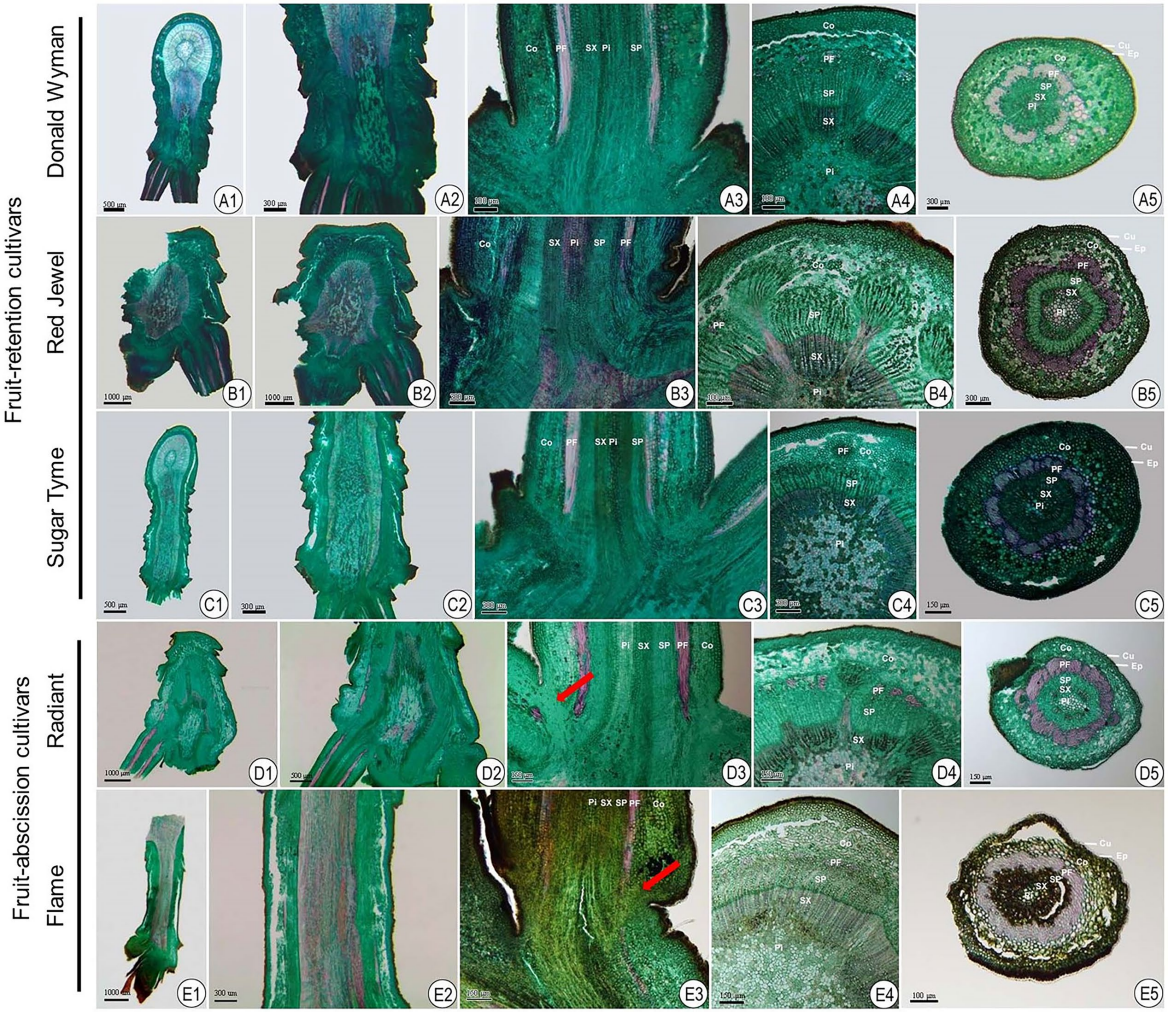
Anatomical observations of fruit AZ in fruit-retention and -abscission cultivars of ornamental crabapple. **(A1–A5)** “Donald Wyman”; **(B1–B5)** “Red Jewel”; **(C1–C5)** “Sugar Tyme”; **(D1–D5)** “Radiant”; **(E1–E5)** “Flame.” **(A1–E1)** and **(A2–E2)** longitudinal section of attached shoot and fruit pedicels; **(A3–E3)** longitudinal section of fruit pedicels; **(A4–E4)** and **(A5–E5)** transverse section of shoot and fruit pedicels, respectively. Cu, Cuticle; Ep, epidermal cells; Co, cortex; PF, phloem fiber; SP, secondary phloem; SX, secondary xylem; and Pi, pith.

Abscission describes the detachment of plant organs from their parent plant. This procedure frequently occurs at the intersection of two distinct organs in one or more AZ-specific cell layers (Yu and Kellogg, 2018). A microtome wood sectioning technique was utilized to examine the architecture histological appearances in longitudinal and transverse orientations of the AZ in ornamental crabapples (Figure 1).

The attachment of fruit pedicel to the dwarf shoot pith (Figures 1A3–E3) was confirmed by longitudinal slices. Additionally, we observed that the internodes of the shoots were exceedingly short. The epidermis, cortex, bast fibers, secondary bast, secondary xylem, and pith constitute shoot and fruit pedicel, in that order, from the inside out (Figures 1A4–E4,A5–E5). The epidermis and cortex together form a protective layer. The secondary bast mostly transports the products of photosynthesis, while the secondary xylem primarily conveys water and ions and provides structural strength. The pith, which is situated in the center of the vascular column, is composed of cells with thin cell walls and storage capacity. In contrast to the shoot, the fruit pedicel’s transverse anatomical structure comprised of the cortex, which, along with the epidermis, served a protective function for the fruit pedicel. In addition, the bast fibers in the fruit pedicel had greatly thicker cell walls, which supported the fruit.

It was further revealed that in fruit-abscission cultivars, the cortical layer at the junction of the fruit pedicel and dwarf shoot had conspicuous microscopic parenchyma cell (Figures 1D3,E3), but in fruit retention cultivars, the cortical cells at the base of the fruit pedicel were homogeneous in size and evenly organization. In these cultivars of fruit-abscission, the abscission layer appears to develop throughout time, indicating that it is the fundamental component of fruit abscission.

### Enzyme activity in the AZ of ornamental crabapple fruits

**Figure 2:**
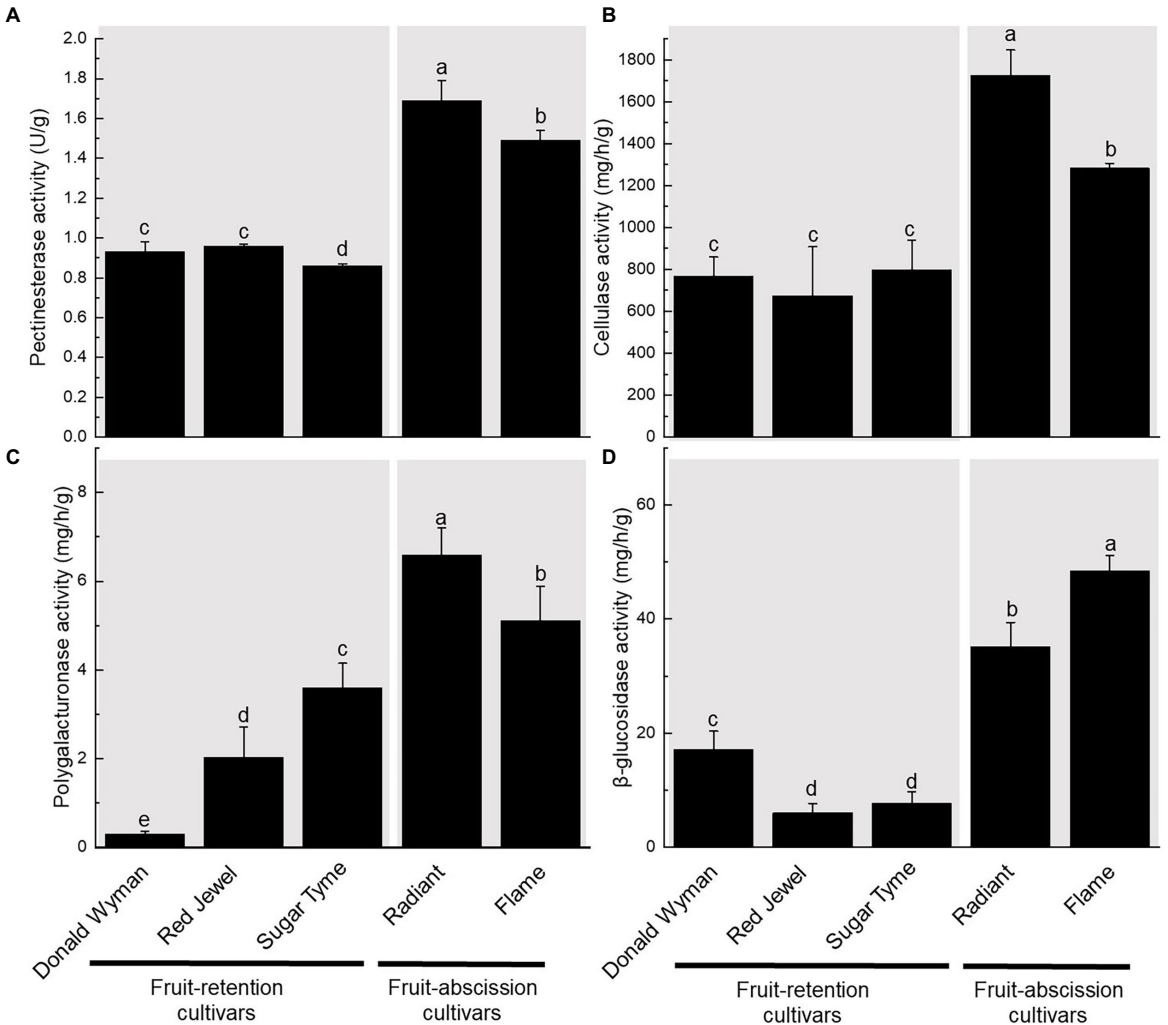
Variation in enzyme activity of fruit pedicels in fruit-retention and -abscission cultivars of ornamental crabapple. **(A)** Pectinesterase (PE); **(B)** Cellulase (CX); **(C)** Polygalacturonase (PG); and **(D)** β-glucosidase (BG). Different letters indicate significant differences at *p* < 0.05.

We measured the amounts of four enzymes (PE, CX, PG, and BG) in five ornamental crabapple cultivars’s dwarf shoot and fruit pedicel (Figure 2). These results demonstrate that fruit-abscission cultivars (“Flame,” “Radiant”) possess significantly higher PE, CX, PG, and BG enzyme activities than fruit-retention cultivars (“Donald Wyman,” “Red Jewel,” and “Sugar Tyme”). The PE and CX enzyme activity of the fruit-abscission cultivars was about 1.5 times that of the fruit-retention cultivars. The activity of PG enzyme was significantly different among the five cultivars, but the enzyme content of the fruit-abscission cultivars was still high. Likewise, we found that the enzyme activity of BG was highest in the “Flame,” which may lead to early abscission of the “Flame” fruits (Figure 2). On the basis of these results, the increased activity of hydrolases (such as PE, PG, CX, and BG) degraded the pectin and cellulose in the cell wall, which may be one of the reasons for the fruit abscission of ornamental crabapples.

### Assembly and annotation of unigenes and differential expression analysis

Using RNA-seq, we sequenced five ornamental crabapple cultivars to explore the transcriptional processes underlying the establishment of the AZ, a determinant in fruit abscission. Transcriptome sequencing results for fruit-retention and fruit-abscission cultivars of ornamental crabapple were shown in Supplementary Table S2.

After quality control and data collection, the total clean data were about 51.59 G. with each cultivar ranging between 6.7 and 10.7 Gb. The GC content ranged from 46.39 to 47.84%, while the Q30 content ranged from 91.79 to 94.60%. A total of 44,830 consensus non-redundant unigenes were generated after assembly, of which 55,196 were longer than 1,000 bp and N50 was 1,807 bp. The total length of all unigenes was 51,673,246 bp. The majority of the unigenes were between 300 and 500 bp in length, accounting for 32.15% of the total. The proportions of lengths between 500 and 1,000 bp, 1,000–2,000 bp, and more than 2,000 bp are 27.75, 23.44, and 16.67%, respectively (Supplementary Table S3).

The annotation of unigenes derived from ornamental crabapples was shown in Supplementary Table S4. It was determined that 33,299 consensus unigenes were annotated, with 16,748 (50.30%) having a length more than 1,000 bp and 16,551 (49.70%) with lengths roughly equivalent to 300 bp but less than 1,000 bp. In the NR database, 32,503 unigenes were annotated, accounting for 97.61% of all annotated consensus unigenes. This is followed by Swiss-Prot (19,643, 59.01%), GO (18,270, 54.87%), KOG (18,090, 54.33%), KEGG (12,922, 38.81%), and COG (11,681, 35.08%).

We used in RNA-seq analysis to study the changes in gene expression during fruit shedding in different ornamental crabapple cultivars, and then revealed the effects of hormones, transcription factors and cell wall hydrolases on fruit abscission. A total of 767 DEGs satisfying the criteria of log_2_(Fold Change) ≥ 1 and FRD < 0.01 was identified between fruit-abscission cultivars and fruit-retention cultivars. Of these, 442 DEGs were upregulated whereas 325 were downregulated.

### Expression levels of phytohormone synthesis-related genes in the fruit abscission transcriptome

**Figure 3:**
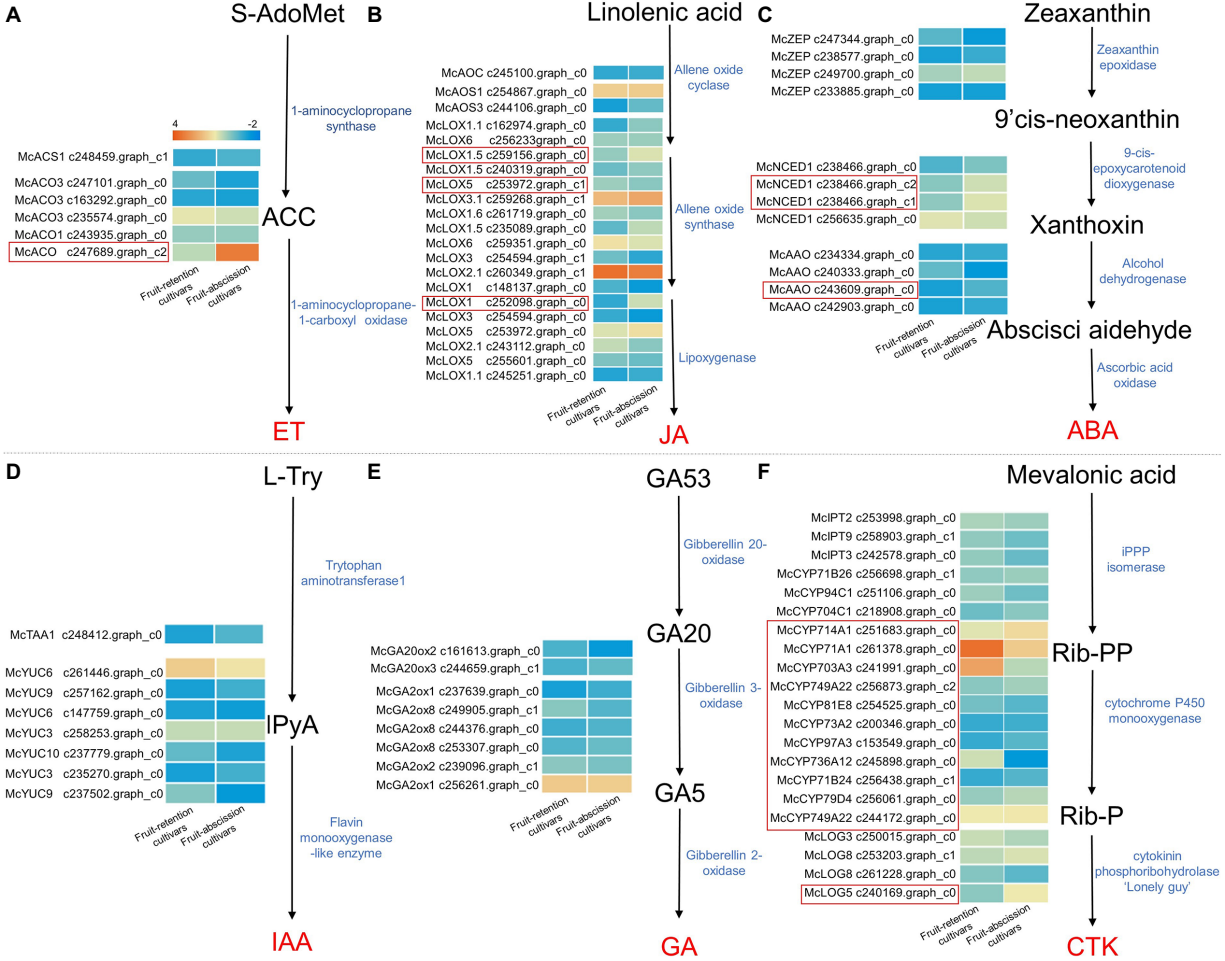
Schematics of biosynthesis differential transcript profiles of hormone-related genes. Genes up- or downregulated in all stages in fruit-retention and -abscission cultivars were denoted by light orange and blue, respectively. **(A)** Ethylene; **(B)** Jasmonic acid; **(C)** Abscisic acid; **(D)** Auxin; **(E)** Gibberellin; and **(F)** Cytokinin.

Hormones govern fruit abscission directly and play a crucial function as a signaling component in the fruit abscission process. To identify potential regulatory mechanisms that leading to the fruit abscission of ornamental crabapple, we compared the transcript levels of key enzymes involved in hormone biosynthesis across fruit-retention and -abscission cultivars (Figure 3). The results revealed that synthetases of six hormone biosynthesis pathways were retrieved, among which ET, jasmonic acid (JA), ABA, and CTK synthesis genes were differentially expressed in cultivars with different fruit abscission types.

Consensus unigenes were used to identify the key synthases *McACS1* and *McACOs* in the ethylene biosynthesis pathway. 1-aminocyclopropane-1-carboxylate oxidase (ACO) is a significant rate-limiting enzyme that plays an essential role in ET biosynthesis regulation (Vanderstraeten et al., 2019). The expression of *McACO* varied significantly across ornamental crabapples with different fruit abscission types (Figure 3A). Similarly, *McAOC*, *McAOS1*, *McAOS3*, and *McLOXs* genes were identified in the JA biosynthesis pathway, the expression levels of *McLOX1, McLOX1.5*, and *McLOX5* differed significantly across fruit-retention and -abscission cultivars (Figure 3B). In the ABA biosynthesis pathway, *McNCED1* and *McAAOs* revealed significantly variable expression levels, while *McZEPs* was not found (Figure 3C). *McTAA1* and *McYUCs* genes, which encode key enzymes in the IAA synthesis pathway, were identified in the fruit’s AZ. However, non-significant differences between their expressions indicate that IAA may not be directly involved in the abscission of ornamental crabapple fruit (Figure 3D). The expression of *McGA20ox2/3* and *McGA2oxs* genes in the GA biosynthesis pathway was not significantly different between the fruit-retention and fruit-abscission cultivars (Figure 3E). For the CTK synthesis pathway, a high number of the *McCYP450s* genes were found with substantial expression differences in different fruit abscission cultivars, but *McIPT2/3/9* and *McLOG3/8* genes showed no significant expression changes (Figure 3F).

In conclusion, the four phytohormones include ET, JA, ABA, and CTK have the potential in the fruit abscission of ornamental crabapple. The considerable differential expression of *McACO*, *McLOX1, McLOX1.5, McLOX5, McNCED1, McAAO, McCYP450s*, and *McLOG5* may have a direct effect on the hormone levels in the AZ. In addition, we hypothesized that IAA and GA have no direct influence on fruit abscission since their synthetic-related genes revealed no significant expression differences. Intriguingly, most of the differentially expressed hormone synthesis genes were upregulated. It is proved that ET, JA, ABA, and CTK may have positive regulatory effects on fruit abscission. Our findings demonstrated the synchronization of multiple hormones that induce fruit abscission in ornamental crabapple.

**Figure 4:**
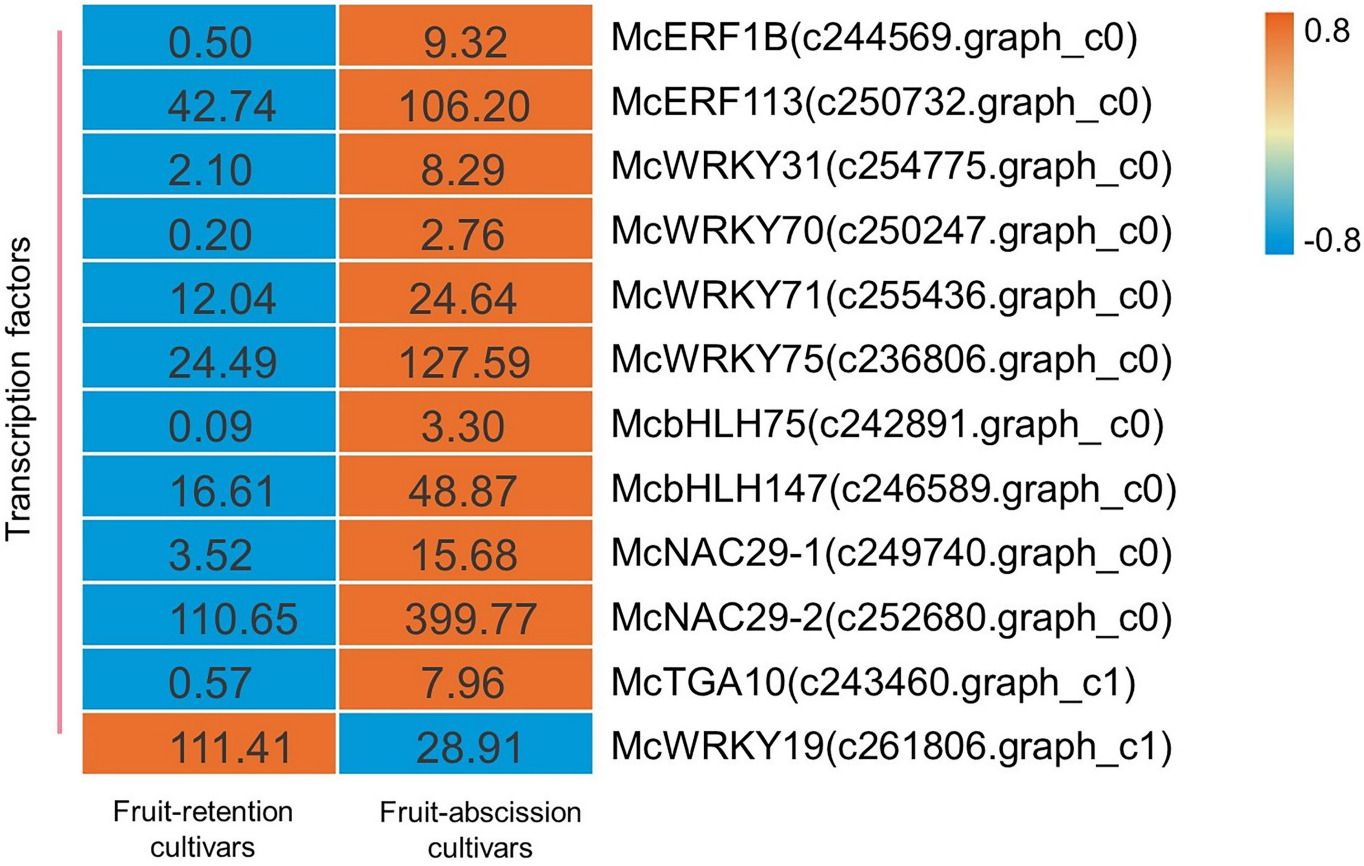
Schematics of biosynthesis differential transcript profiles of hormone metabolism-related genes and TFs. Genes up- or downregulated in all stages in fruit-retention and -abscission cultivars are denoted by orange and blue, respectively.

There is a crucial function for transcription factors throughout every phase of a plant’s development (Jiang et al., 2020). Transcriptome data were used to identify the transcription factors (TFs) involved in the regulation of ornamental crabapple fruit abscission, and to explore the regulatory mechanism of phytohormones on signal transduction. Some TFs potentially regulated by hormones were analyzed, and their expression varied across ornamental crabapple cultivars. There were identified 12 TFs including family ERF, WRKY, bHLH, NAC, and TGA. Finally, a heatmap depicting the expression levels of these TFs was shown (Figure 4).

Numerous studies have shown that the aforementioned transcription factor families are connected with the shedding of plant organs. The AP2/ERF family of transcription factors, *RhERF1* and *RhERF4*, has a function in the abscission of petals in Rosa hybrida (Gao et al., 2019). After melon fruit ripens, WRKY transcription factors are activated and engaged in fruit abscission (Corbacho et al., 2017). *CitbHLH1*, a member of the transcription factor family involved in the function of signaling hormones including ET and JA (Sun et al., 2018), may also play a significant role in regulating fruit abscission (Agusti et al., 2012). Likewise, NAC TFs in litchi may be associated with the transcriptional cascade initiated by ET-driven fruit abscission (Li et al., 2015). In addition, TGA TFs in *Arabidopsis* contributed to the establishment of a BOP-dependent AZ, which resulted to the abscission of petals (Corrigan, 2018). Several TFs linked with abscission, including ERF (*McERF1B* c244569.graph_c0, *McERF113* c250732.graph_c0), WRKY (*McWRKY31* c254775.graph_c0, *McWRKY70* c250247.graph_c0, *McWRKY71* c255436.graph_c0, and *McWRKY75* c236806.graph_c0), bHLH (*McbHLH75* c242891.graph c0, *McbHLH147* c246589.graph_c0), NAC (*McNAC29-1* c249740.graph_c0, *NAC29-2* c252680.graph_c0), and TGA (*McTGA10* c243460.graph_c1), were upregulated in our research. Furthermore, *McWRKY19* (c261806.graph_c1) was considerably down-regulated in fruit-abscission cultivars. This finding highlighted the function of TFs in the fruit abscission of ornamental crabapple and shed light on the underlying molecular process.

### Transcriptome expression trends of plant cell wall hydrolase-related synthetic genes in different fruit abscission types

**Figure 5:**
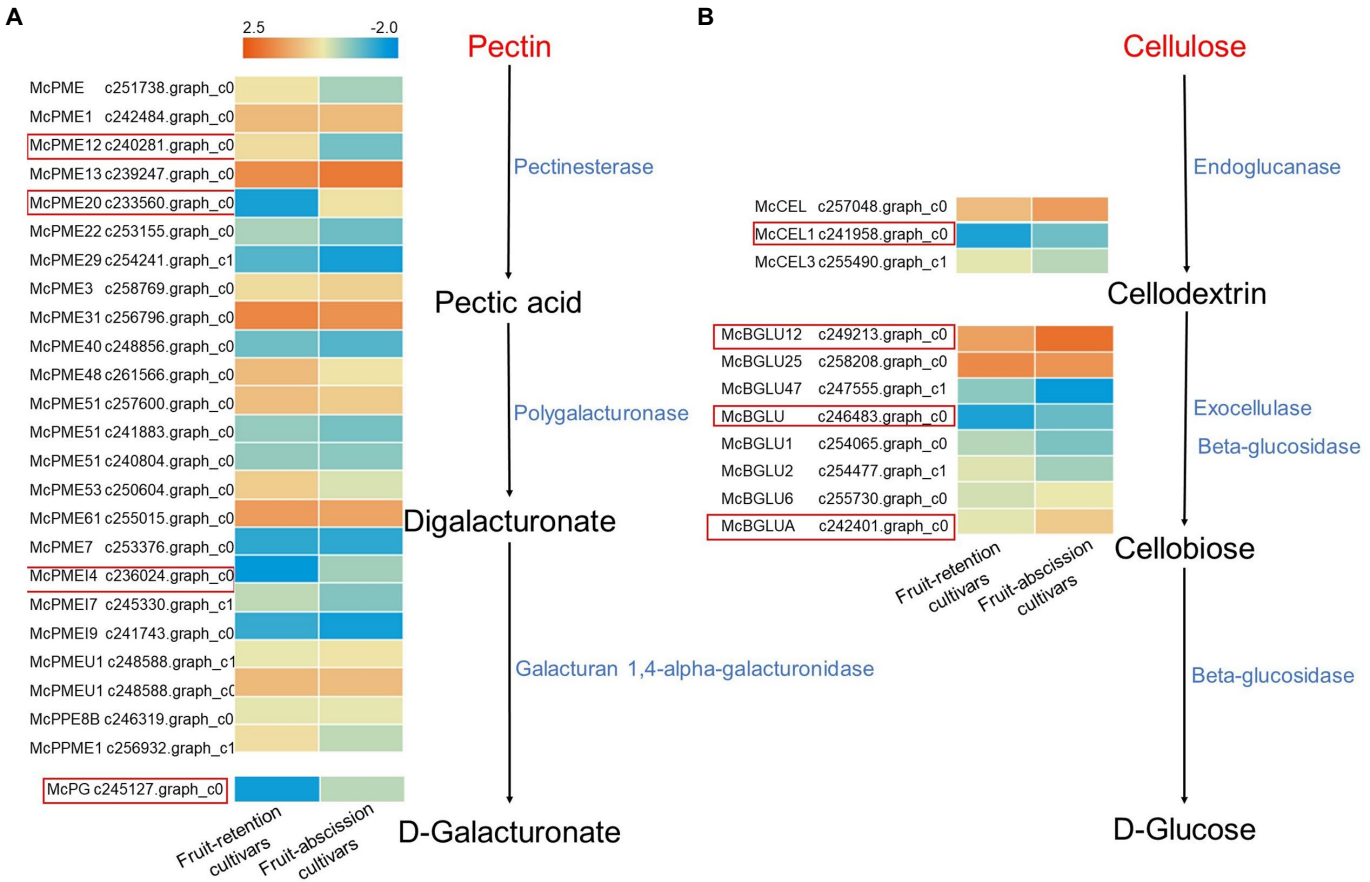
Schematics of metabolic differential transcript profiles of cellulose and pectin synthesis related genes. Genes up- or downregulated in all stages in fruit-retention and -abscission cultivars are denoted by light orange and blue, respectively. **(A)** Pentin; **(B)** Cellulose.

Pectin and cellulose are the main components of plant cell walls, they sustain the entire cell structure and prevent its degeneration. Pectin in the abscission layer was dissolved during fruit abscission by hydrolases such as CX and PG, which weakens the cell wall and result in fruit abscission (Gulfishan et al., 2019). To further explore the enzymes involved in the degradation of abscission cells, we identified the enzyme-encoded genes in the metabolic pathway of pectin and cellulose in the transcriptome (Figure 5). *McPMEs* (c240281.graph_c0, c233560.graph_c0, c236024.graph_c0), *McPG* (c245127.graph_c0), *McCEL1* (c241958.graph_c0), and *McBGLUs* (c249213.graph_c0, c246483.graph_c0, c242401.graph_c0) were significant expressed in the AZ pectin and cellulose metabolic pathway. Other enzyme-encoded genes were not significantly varied among fruit-retention and -abscission cultivars. The findings confirmed that the buildup of PE and CX in the AZ facilitated the fruit abscission of ornamental crabapple.

### Expression validation of differentially expressed cell wall hydrolases and TFs in the transcriptome

**Figure 6:**
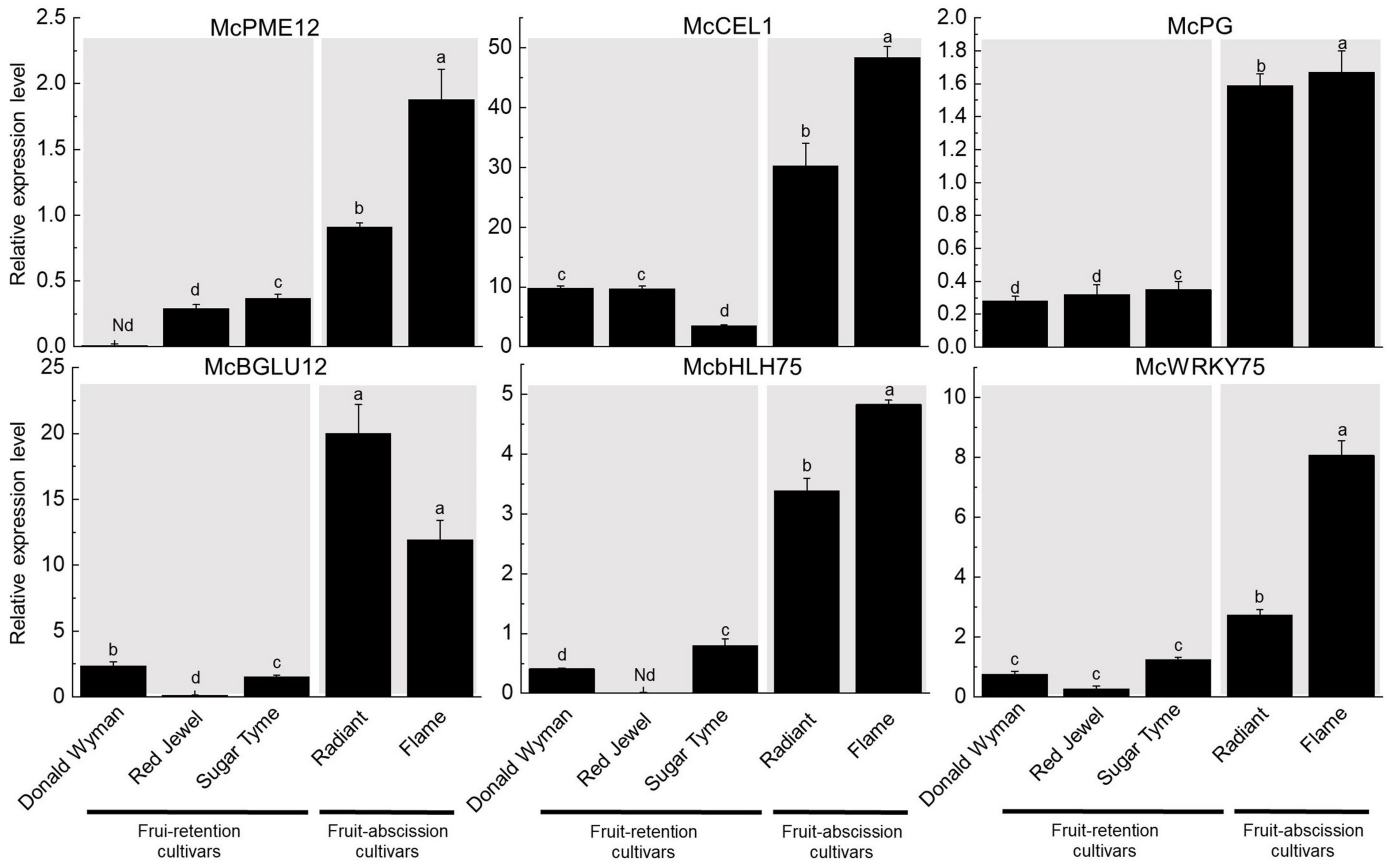
Six genes were selected for quantitative real-time PCR (qRT-PCR). The data were normalized by using McActin as an internal reference. Data were represented as mean ± SD for three biological replicates.

To verify the differential expression of cell wall hydrolases and TFs in various abscission type cultivars, six genes were selected: four cell wall hydrolases (*McPME12*, *McCEL1*, *McBGLU12*, and *McPG*) and two TFs (*McbHLH75*, *McWRKY75*). The elevated expression levels of *McPME12*, *McCEL1*, *McBGLU12*, and *McPG* in fruit-abscission cultivars relative to fruit-retention cultivars indicate the importance of cell wall hydrolases in fruit abscission. In addition, the increased expression of *McbHLH75* and *McWRKY75* in fruit-abscission cultivars provides more evidence for the function of TFs in fruit abscission. The identification of expression levels further confirmed the reliability of the transcriptome data (Figure 6).

**Figure 7:**
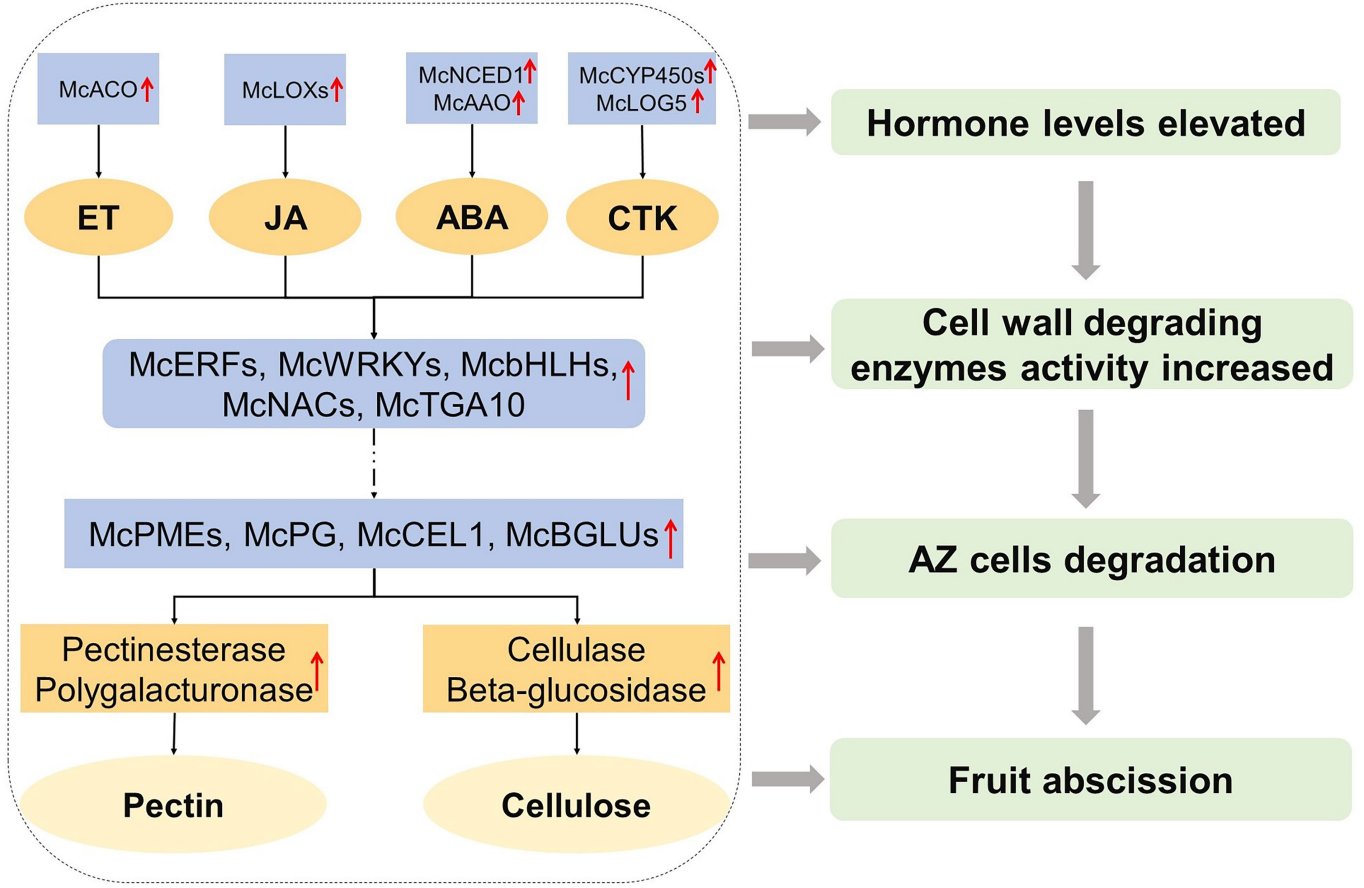
A model for regulating fruit abscission in ornamental crabapple was proposed. The expression of ethylene biosynthesis genes, jasmonic acid biosynthesis genes, abscisic acid biosynthesis genes, and cytokinin biosynthesis genes upregulated in AZ during fruit abscission. Cell wall hydrolase-related genes were also upregulated. Solid arrows indicate known mechanisms; broken arrows indicate unknown mechanisms, and red represents upregulation increase events.

According to the differences in the expression levels of the above related genes, the response hormones of different abscission cultivars of ornamental crabapple were determined, and finally the abscission was successful. Therefore, we constructed a fruit abscission model of ornamental crabapple to demonstrate the possible internal mechanism affecting fruit abscission of ornamental crabapple (Figure 7).

## Discussion

Fruit abscission is a normal physiological phenomenon that is mostly triggered by internal factors. Here, analyzing fruit pedicel length, fruit pedicel thickness, and fruit quality of fruit-retention and -abscission cultivars, we found that the fruit weight of “Radiant” was 1.05 g; the weight of “Flame” was 3.42 g. Interestingly, both “Flame” with the large weight fruit and “Radiant” with the small weight fruit were found that be shedding varieties in our study. Previous research has shown that the small fruit of the citrus variety is late shedding (Ruiz et al., 2001). In citrus, late shedding of small fruit is influenced by carbohydrate supply limitation, and it is speculated that free sugar levels in the peel may be the signal that triggers shedding. However, according to our article, “Radiant” is early shedding variety. These suggested that fruit weight may not be the major determinants in early fruit abscission of ornamental crabapple. The fruit drop of ornamental crabapple may most due to internal factors.

Previous studies have shown a clear correlation between fruit abscission and plant cell structure alterations. During the berry shedding process of different grape varieties, the separation layer’s cells expand in volume and become progressively hydrolyzed, or liquefied (Li et al., 2020). PE, PG, CX, and BG affect abscission by degrade the AZ and changing the cell structure (Phetsirikoon et al., 2016; Patharkar and Walker, 2019). The activity of PGs is significantly elevated in the side of secondary xylem where the fruit is attached to the receptacle (Tabuchi et al., 2000). *CsPME1* and *CsPME3* in Valencia orange were differentially expressed and influenced fruit abscission (Joseph et al., 1998). PGs were also seen in the distal and proximal ends of the tomato pedicel explants during the entire shedding process. In tomato, PG is associated with pedicel abscission based on its distribution in isolated areas (Qi et al., 2014). After induction of pedicel shedding, two PG genes, *TAPG1* and *TAPG4*, were more expressed in proximal AZ cells than in distal AZ cells (Kalaitzis et al., 1997). During litchi fruit drop, cell wall degradation and cell separation occurred, as well as an increase in cellulase activity and a reduction in cellulose content. Its two CEL genes (*LcCEL2* and *LcCEL*8) are closely related to shedding (Li et al., 2019). In the calyx ablating region of Valencia orange, the activities of endogenous -1, 4-β-glucanase (cellulase) and polygalacturonidase were dramatically increased (Burns et al., 1998). In this research, fruit-abscission cultivars exhibited significant alterations in PE, CX, PG, and BG. This is roughly consistent with the results of previous studies.

Phytohormones control the fruit abscission. According to their function, different hormones may be split into two categories: one promotes aging, shedding and inhibit growth, while the other inhibits aging and promotes growth. According to prior study, ET can accelerate abscission formation and enhance organ shedding (Bunya-atichart et al., 2011; Meng et al., 2019). In apple, the increase of *MdACO1* (constructional triple reaction 1) was detected in fruit separations (Botton et al., 2011). In litchi, *LcEIL2/3* genes activated *LcCEL2/8*, *LcPG1/2* (cell wall remodeling genes) and *LcACS1/4/7*, *LcACO2/3* (ether biosynthesis genes) through interaction with promoters to affecting the process of fruit shedding (Ma et al., 2020). The expression of *McACO* in our research was significantly increased in fruit-abscission cultivars compared to fruit-retention cultivars, as shown by transcriptome analysis. This further demonstrates the effect ET in fruit abscission, although its precise mechanism requires more research.

Jasmonic acid also has a significant influence in fruit shedding. Methyl jasminate (MeJA) can affect moss’s internode growth by interacting with IAA. Furthermore, MeJA can induce the formation of secondary AZs in bryophytes by altering endogenous levels of auxin and JA-related compounds (Dziurka et al., 2022). Simultaneously, MeJA, JA, and 1-aminocyclopropane-1-carboxylic acid (ACC) were administered to grape berries, revealing that ACC could promote MeJA-induced grape shedding (Fidelibus et al., 2022). These findings also indicated that ET and JA may have a synergistic effect on fruit abscission regulation. In this work, JA synthesis genes *McLOX1*, *McLOX1.5*, and *McLOX5* in fruit-abscission cultivars were significantly upregulated. It is shown that JA affected fruit shedding. However, additional research is required to determine if JA and ET may act together to regulation fruit abscission in ornamental crabapple.

Abscisic acid and CTK signaling also play vital roles in the process of fruit abscission. *McNCED1* and *McAAO*, two ABA synthase genes, were identified to be up-regulated in fruit-abscission cultivars of ornamental crabapple. Previous research also showed that *LgAAO3* and *LgNCED* genes were upregulation during longan fruit drop, which resulted from the accumulation of ABA in the AZ (Wei et al., 2021). In addition, the ABA is abundant while CTK is low in litchi fruit, which may influence fruit shedding (Qiu et al., 1998). During rose petal shedding, the *RbLOG* gene, which encodes the cytokinin nucleoside 5′-monophosphate phosphoribosyl hydrolase, is upregulated (Singh et al., 2020). Results from this study’s transcriptome analysis also revealed that differential expression of *McLOG5*, indicating that CTK may play crucial roles in the fruit abscission process of ornamental crabapple. ET may interact with other hormones, such as ABA and CTK (Meir et al., 2010). In fact, once the mechanisms associated with non-dropping fruit are triggered, it seems that the molecular process of both ET and ABA biosynthesis are considerably and rapidly downregulated (Vriezen et al., 2008; Nitsch et al., 2009). Evidently, ET, JA, CTK, and ABA may play a role alone or in concert in the process of ornamental crabapple fruit shedding, and the specific mechanism needs to be explored further.

Transcription factors are considered to be the main switches regulating the cascade in development and various biological processes (Riechmann et al., 2000). During tomato’s fruit abscission, ethylene responsiveness factor 52 (*SiERF52*) acts as a central TF to activating CELs and PGs (Nakano et al., 2014). In this study, we also found that *McERF1B*, *McERF113* in ornamental crabapple were upregulated in fruit-abscission varieties. Our findings supported the conclusions of previous research that ET induces fruit abscission. The regulatory action of ET on fruit abscission, which may be mediated through the activation of ERF transcription factors to govern the establishment of the AZ in the fruit pedicel, requires more elaboration. We also discovered that several WRKY transcription factors had significantly variable expression levels in different cultivars of ornamental crabapple. Among them, *McWRKY31/70/71/75* was upregulated while *McWRKY19* was downregulated. This discrepancy in expression pattern may be a result of the distinct transcriptional regulatory functions of WRKY transcription factors. During ET-induced fruit abscission of litchi, the expression levels of *LcbHLH* and *LcNAC* transcription factors in AZ were altered (Li et al., 2015). Similar to the prior findings, we found that the expression levels of bHLH (*McbHLH75/147*) and NAC (*McNAC29-1*, *McNAC29-2*) transcription factors were different in ornamental crabapple with distinct in fruit-abscission cultivars. It is also worth noting that *McTGA10* is a member of the bZIP transcription factor. TGA transcription factor can bind to downstream genes specifically to regulate fruit shedding of *Phaseolus vulgaris* (Sriskantharajah et al., 2021). Therefore, the differential expression of *McTGA10* was anticipated to mediate changes in other genes that affect the fruit abscission of ornamental crabapples. Due to the variable endogenous hormone concentrations in the AZ, different ornamental crabapple cultivars expressed by a large number of TFs in a manner that were distinct from one another. These TFs may stimulate the synthesis of cell wall hydrolases in the fruit AZ of ornamental crabapple.

Following the conclusion of the preceding research, we put forth a hypothesized model in an effort to regulate the fruit abscission of ornamental crabapple (Figure 7). In this paradigm, ET, JA, ABA, and CTK are the primary signaling hormones, and their synthesis genes are upregulated to alter the internal hormone levels. Those phytohormone-related downstream TFs, *McERFs*, *McWRKYs*, *McbHLHs*, *McNACs*, and *McTGA10*, were boosted and played a signal transduction function, collaborating with their downstream genes to increase the expression of cell wall hydrolase production genes (*McPMEs*, *McPG*, *McCEL1*, and *McBGLUs*). Subsequently, the activity of cell wall hydrolases (pectinesterase, polygalacturonase, cellulase, and β-glucosidase) was enhanced to finally degrade the cells in the AZ, resulting in the abscission of ornamental crabapple fruit.

## Data availability statement

The data presented in the study are deposited in the Genome Sequence Archive repository, accession number CRA004670.

## Author contributions

GZ and XW conceived the study. XW, SY, XS, HL, BC, and XX collected materials and performed experiments. XW, YW, and ZW analyzed the RNA-seq data and drafted the manuscript. XW, YW, ZW, and GZ revised the manuscript. All authors contributed to the article and approved the submitted version.

## Funding

This work was supported by the Hebei Provincial Department of Science and Technology Project (21326301D), National “Twelfth Five-Year” Plan for Science and Technology Support (2012AA102002-5) and Hebei University Horticultural Crop Breeding Application Technology R & D Center (YF201404). The funders had no role in study design, data collection and analysis, decision to publish, or preparation of the manuscript.

## Conflict of interest

The authors declare that the research was conducted in the absence of any commercial or financial relationships that could be construed as a potential conflict of interest.

## Publisher’s note

All claims expressed in this article are solely those of the authors and do not necessarily represent those of their affiliated organizations, or those of the publisher, the editors and the reviewers. Any product that may be evaluated in this article, or claim that may be made by its manufacturer, is not guaranteed or endorsed by the publisher.

## Supplementary material

The Supplementary material for this article can be found online at: https://www.frontiersin.org/articles/10.3389/fpls.2022.1013263/full#supplementary-material

SUPPLEMENTARY FIGURE S1 Comparison of the different period of ornamental crabapple fruit. **(A)** Show the changes of fruit growth status of five cultivars of ornamental crabapple from August to November. Fruit abscission fell off completely in October **(B)**. The location of fruit-retention cultivars and the location of fruit-abscission cultivars.
