# Supplementary figures and images for: A multifaceted comparison between the fruit-abscission and fruit-retention cultivars in ornamental crabapple
Source: Front Plant Sci. 2022 Sep 21;13:1013263. doi: 10.3389/fpls.2022.1013263 (PMC9535355; doi:10.3389/fpls.2022.1013263)

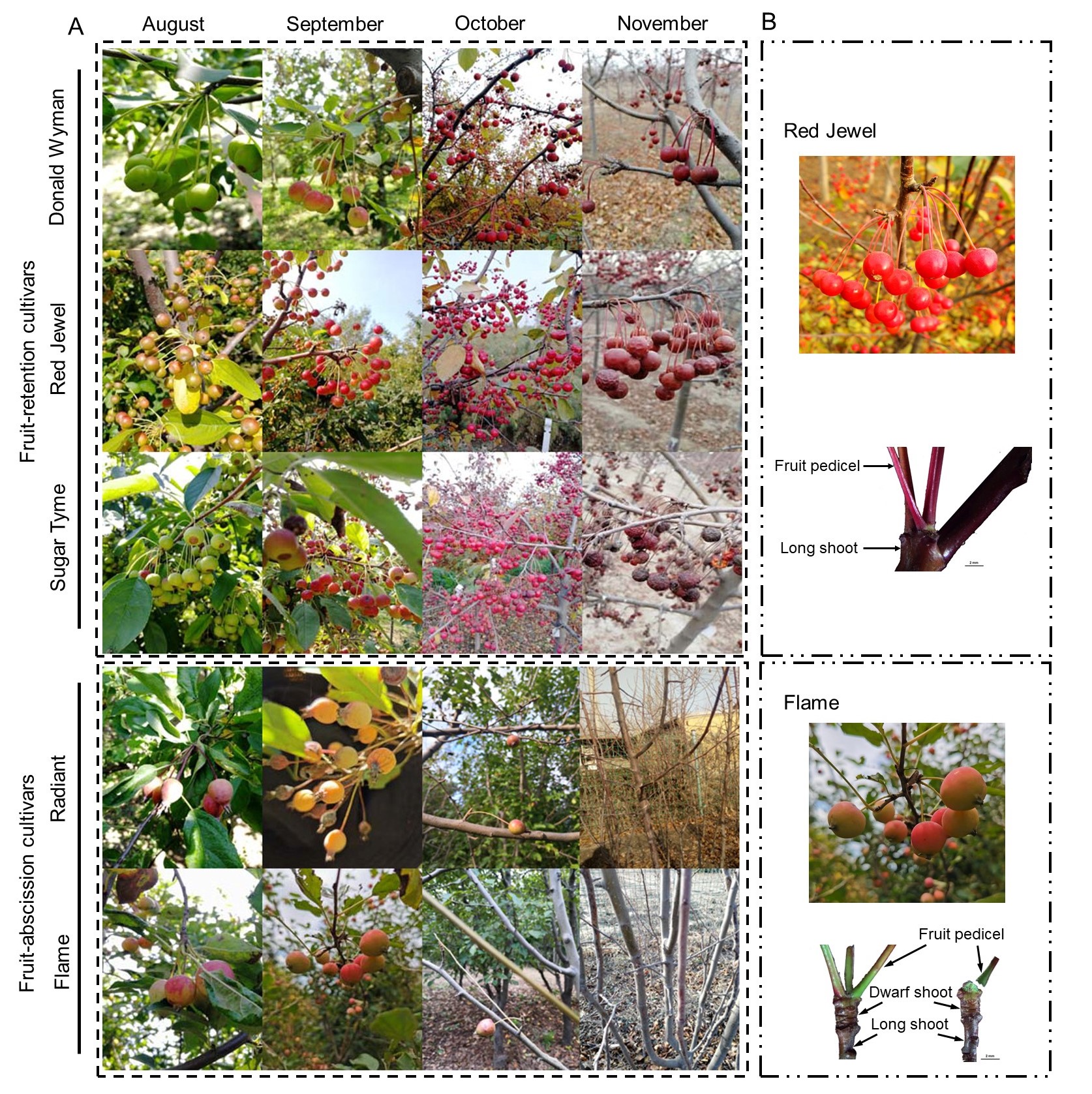

Supplement: SUPPLEMENTARY FIGURE S1 — Comparison of the different period of ornamental crabapple fruit. (A) Show the changes of fruit growth status of five cultivars of ornamental crabapple from August to November. Fruit abscission fell off completely in October (B). The location of fruit-retention cultivars and the location of fruit-abscission cultivars. [file Image_1.jpg]
